# HIV-1 infection of renal epithelial cells: 30 years of evidence from transgenic animal models, human studies and in vitro experiments

**DOI:** 10.1186/s12977-023-00617-8

**Published:** 2023-03-16

**Authors:** Maria Blasi, Mary Klotman

**Affiliations:** 1 Department of Medicine, Division of Infectious Diseases, Duke University School of Medicine, Durham, NC USA; 2 Duke Human Vaccine Institute, Duke University School of Medicine, Durham, NC USA; 3 Duke University Medical Center, MSRBII Room 3077, Durham, NC 27710 USA; 4 Duke University Medical Center, 2927, Davison Building Room 125, Durham, NC 27710 USA

**Keywords:** HIV-1, Kidney, Reservoir, Renal epithelial cells, Urine

## Abstract

Although antiretroviral therapy (ART) has increased life expectancy in people with HIV-1 (PWH), acute and chronic kidney disease remain common in this population and are associated with poor outcomes. A broad spectrum of kidney disorders can be observed in PWH, some of which are directly related to intrarenal HIV infection and gene expression. HIV-associated nephropathy (HIVAN) was the most common kidney disease in PWH before ART became available. Animal models and human biopsy studies established the causal relationships between direct HIV-1 infection of renal epithelial cells and HIVAN, expression of viral genes in renal epithelial cells, and dysregulation of host genes involved in cell differentiation and cell cycle. In this review, we provide a summary of the body of work demonstrating HIV-1 infection of epithelial cells in the kidney and recent advancements in the understanding of viral entry mechanisms and consequences of HIV-1 gene expression in those cells.

**Graphical Abstract:**

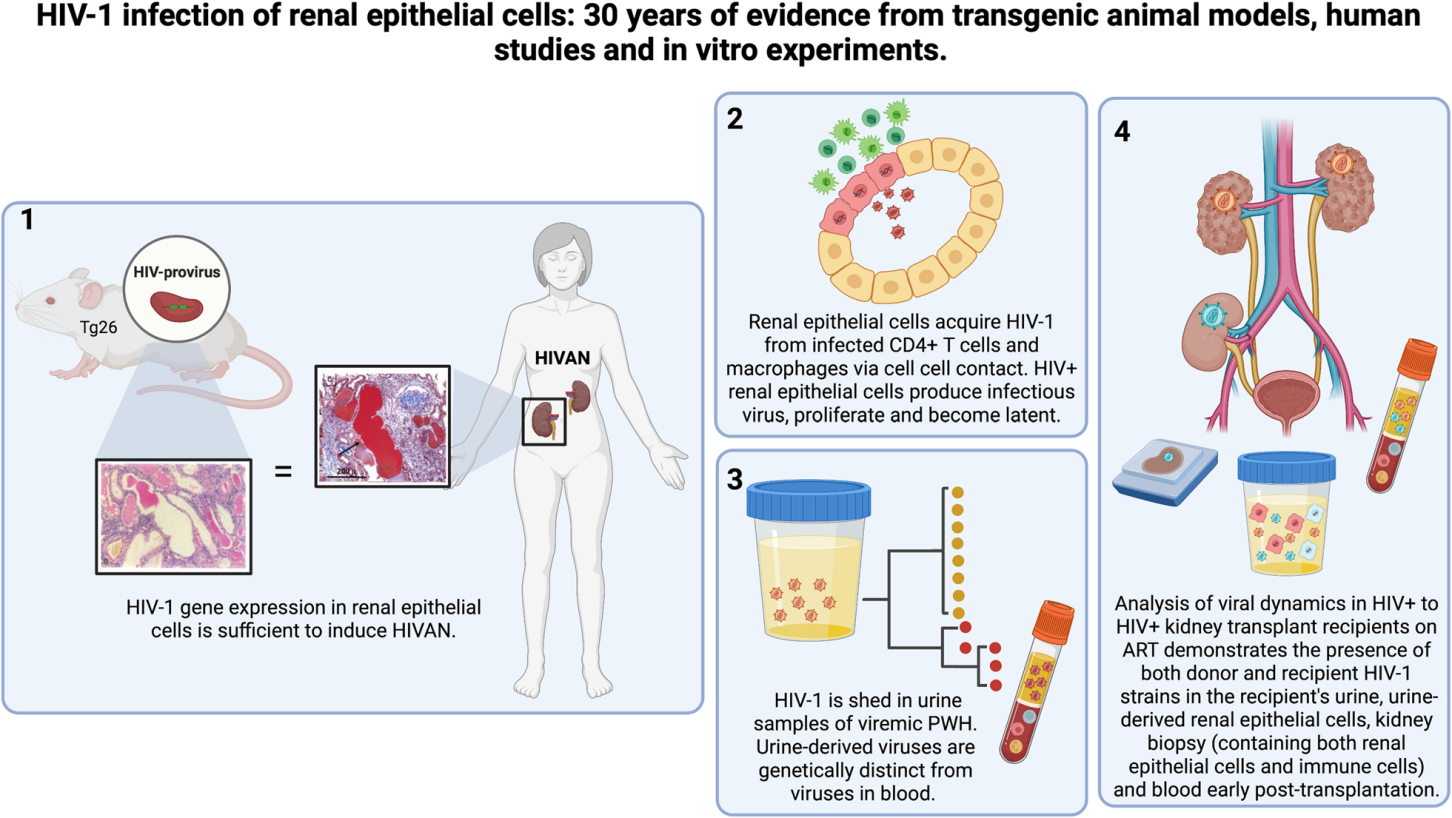

## Background

Antiretroviral therapy (ART) effectively blocks HIV-1 replication and prevents transmission, but it does not eradicate HIV-1 from the body of infected individuals. HIV-1 has been shown to persist in multiple anatomical reservoirs during therapy [1], from which it can reactivate when therapy is interrupted. The persistence of HIV-1 in different tissues throughout the body of infected individuals has important implications for viral pathogenesis and represents the main barrier to achieving an HIV-1 cure. HIV-1 has been detected in nearly every tissue that has been analyzed [2]. In this review we will focus on the evidence demonstrating HIV-1 infection in kidney epithelial cells and will provide an overview of human, animal and in vitro studies demonstrating HIV-1 infection, replication, latency and persistence in renal epithelial cells.

## Transgenic animal model of renal HIV-1 infection and studies on human kidney tissue demonstrating HIV-1 infection and gene expression in renal epithelial cells

**Fig. 1:**
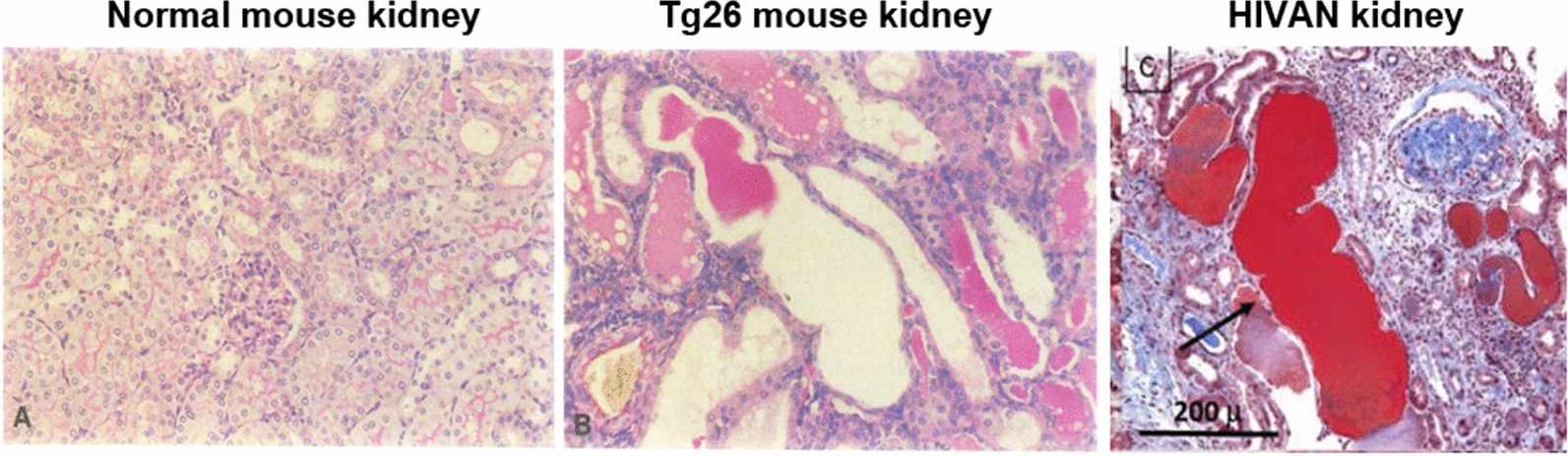
Comparison of renal histology in a normal mouse, a Tg26 mouse and an HIVAN kidney biopsy. The pathology in the Tg26 kidney mirrors HIVAN kidney pathology, including focal segmental glomerulosclerosis (FSGS), microcystic tubular dilatation and monocytic infiltrate. Adapted from Kopp et al. [4]

People with HIV-1 (PWH) have increased risk for both acute and chronic kidney disease. The classic kidney disease in untreated HIV-1 infection is called HIV associated nephropathy (HIVAN) and is characterized by proteinuria, renal dysfunction, tubules dilatation and interstitial inflammation [3]. The association between HIV-1 infection of renal epithelial cells and HIVAN was first demonstrated in 1992 using a transgenic mouse model of renal HIV-1 infection called Tg26 [4]. In this mouse model the expression in kidney epithelial cells of a proviral HIV-1 genome lacking a 3 kb sequence overlapping the gag and pol genes, induced proteinuria, severe nephrotic syndrome, and rapid progression to end-stage renal failure. As shown in Fig. 1 the renal histology of Tg26 mice demonstrated focal segmental glomerulosclerosis (FSGS) and microcystic tubular dilatation, mirroring the pathology observed in human HIVAN tissues. These results demonstrated that HIV-1 integration into kidney epithelial cells contributes to tissue injury even when systemic viral replication is absent.

Following these earlier studies in the Tg26 mouse model, several reports from our group and others reported the presence of HIV-1 in renal epithelial cells of people with HIVAN. In a study published in 2000, our group detected the presence of HIV-1 nucleic acids in human kidney biopsies by in situ hybridization and in situ PCR [5]. A comparison between kidney tissue from two seropositive individuals, one that developed HIVAN and one that didn’t demonstrate that in the non-HIVAN tissue few HIV-1 positive cells, likely leukocytes, could be detected in the interstitium, while in the HIVAN tissue several HIV-1 positive renal tubules could be observed [5]. Interestingly, it was also noted that when an HIV-1 positive renal tubule was detected in the kidney, often all the epithelial cells lining that tubule contained HIV-1, suggesting either virus spread between neighboring cells or clonal proliferation of HIV-1 infected renal epithelial cells. This latter hypothesis will be discussed further below.

In a follow up study, our group amplified and genetically characterized HIV-1 sequences in renal tubule epithelial cells isolated from kidney biopsies by laser-capture microdissection [6]. The phylogenetic analysis of those sequences demonstrated viral compartmentalization in the kidney [6], suggesting independent HIV-1 replication and evolution in renal epithelial cells.

An independent study by Canaud et al., conducted on people with HIV-1 that received a kidney from HIV-1 negative donors demonstrated that HIV-1 reinfected the allografted kidney in 68% of the transplant recipients despite undetectable plasma viremia [7]. In this study HIV-1 DNA and RNA were detected both in podocytes and renal tubule epithelial cells. The presence of HIV-1 in podocytes was associated with podocyte apoptosis and more rapid decline in kidney function, while infection of renal tubule epithelial cells was associated with tubulointerstitial inflammation [7]. Whether HIV infection of the kidney allograft after implantation contributes to reduced long-term allograft survival rates in HIV+ transplant recipients is currently unknown [8].

## HIV-1 shedding and compartmentalization in urine samples of PWH with and without kidney disease

HIV-1 replication in different tissues generates distinct viral populations, viral compartments, and reservoirs [9–11]. A major limitation to studying the kidney as a site of viral replication and persistence in PWH is the inability to collect fresh kidney tissue without a clinical indication for a biopsy, and even when a kidney biopsy is performed, the amount of tissue collected is very limited and only representative of a small portion of the kidney. Examination of the urine offers an opportunity to look at viral particles and/or renal epithelial cells and therefore we optimized protocols to isolate and genetically characterize HIV-1 in urine supernatants and urine-derived renal epithelial cells collected from PWH to determine whether urine could be used as a surrogate marker for renal HIV-1 infection [12, 13]. In addition to demonstrating the presence of genetically distinct and compartmentalized viruses in urine [12–15], our studies demonstrated the presence of several identical HIV-1 sequences across urine samples collected longitudinally [12, 15], suggesting that the source of those urine-derived sequences may be a population of clonally expanded cells in the genitourinary tract, similar to what has been described for infected T cells in the bloodstream [16, 17]. Importantly, HIV-1 RNA can only be detected in urine supernatants from PWH with a detectable plasma viral load (not on ART) [12], suggesting that ART is efficient at suppressing viral replication and preventing viral shedding from this compartment.

Although the detection of distinct viral populations in urine indicated the presence of a distinct viral compartment in the genitourinary tract, these studies did not prove that those viruses were coming from kidney epithelial cells. In more recent work conducted by our group on HIV+ kidney transplant recipients receiving a kidney from HIV+ donors under the HIV Organ Policy Equity (HOPE) Act [18], we have been able to compare viral sequences between urine supernatants, renal epithelial cells cultured from urine and kidney biopsies [13]. We found donor virus in urine and urine-derived renal epithelial cells of a HOPE Act kidney transplant recipient up to 2 weeks post-transplantation despite continuous ART [13]. Few viral sequences corresponding to the donor HIV-1 strain were also detected in the recipient blood, but only at day 3 post-transplantation. In addition, several HIV-1 sequences were also amplified from a kidney-biopsy taken from the donor kidney before implantation, which further indicated HIV-1 infection in the allograft. The analysis of HIV-1 *Env* sequences from renal epithelial cells cultured from the recipient’s urine samples obtained both before and after transplantation revealed that among the sequences amplified from urine collected soon after transplantation, 40% corresponded to the recipient’s HIV-1 strain and 60% to the donor’s strain, a finding that supports the presence of HIV-1 infected renal epithelial cells in both the native and transplanted kidneys. Renal cell–derived sequences were closely related to those amplified from cell-free HIV-1 RNA in urine supernatants and were compartmentalized from blood-derived sequences [13], which supports renal epithelial cells as a source of cell-free virus in such samples. Since this first report, we enrolled additional HOPE Act kidney transplant recipients from which we continue to collect longitudinal samples. In line with our previous reports [12, 15], we have been able to detect cell-free donor-derived HIV-1 RNA sequences in urine supernatants and blood plasma only in the transplant recipients that received a kidney from a viremic donor (unpublished data). However, donor HIV-1 nucleic acids were amplified in the kidney biopsies taken before organ implantation in several recipients, including three where the donor had undetectable plasma viral load (unpublished data). Both donor and recipient HIV-1 strains have also been amplified in urine-derived renal epithelial cells from multiple recipients (unpublished data). Whether the detection of HIV-1 nucleic acids in the donor and native kidneys translates to long-term HIV persistence in the kidneys and potential viral reactivation in the recipient is currently under investigation.

## How does HIV-1 enter kidney cells?

Renal epithelial cells do not express the canonical HIV-1 receptors CD4, CCR5 and CXCR4 found on CD4+ T cells or macrophages. This raises the question of how HIV-1 enters those cells. In the earlier studies mentioned above demonstrating the presence of HIV-1 in renal epithelial cells of HIVAN tissues, it was noted that in addition to infected renal epithelial cells, there were also abundant infected leukocytes in the interstitium [5], which led us to hypothesize that the virus is transferred to renal epithelial cells by infected CD4+ T cells and macrophages that come into direct contact. Cell to cell transfer is a common mechanism used by retroviruses to move from one cell to another, avoiding contact with neutralizing antibodies [19–21]. We initially reported that co-cultivation of HIV-infected T cells with noninfected renal tubular epithelial cells results in a considerable transfer of viral material to the renal epithelial cells through a CD4− and HIV-1 Envelope (Env)-independent mechanism [22]. In a follow-up study we demonstrated that renal tubule epithelial cells are productively infected by HIV-1 and multiple copies of HIV-1 per cell can be transferred from infected T cells to renal epithelial cells [23]. We also showed that persistent expression and generation of infectious virus in renal epithelial cells requires HIV-1 integration, and that co-cultivation of HIV-1-infected renal epithelial cells with non-infected T cells resulted in HIV-1 transmission to T cells, supporting a bidirectional exchange of virus between T cells and kidney-derived epithelial cells [23]. In a more recent study we demonstrated that HIV-1 infected macrophages are also capable of transferring virus to renal tubule epithelial cells in a contact dependent manner [24].

## Viral genes expression and renal epithelial cell fates

Expression of individual viral genes in renal epithelial cells is sufficient to induce pathogenic changes in these cells [25]. Expression of HIV-1 Nef results in podocyte proliferation [26] and dedifferentiation [27] while expression of HIV-1 Vpr induces apoptosis, mitotic cell cycle arrest, polyploidy and hypertrophy both in vitro [28, 29] and in a inducible tubular epithelial cell-specific Vpr transgenic mouse model [30]; all reminiscent of tubular injuries observed in HIVAN. Furthermore, in the Vpr-transgenic mouse model, expression of Vpr in the distal convoluted tubule contributes to renal sodium transport dysregulation, a phenotype commonly observed in hospitalized PWH [31]. These two viral proteins are sufficient to induce tissue damage, and they work synergistically when co-expressed in the same cell, leading to more severe nephropathy [32].

In addition to abnormal pathogenic cellular phenotypes, a recent study from our group demonstrated that individual HIV-1 infected renal epithelial can proliferate and generate expanded cell clones in vitro [24], providing a potential mechanism by which HIV-1 infection may persist and propagate within the kidney, similarly to what has been demonstrated for other cell types [33]. As mentioned above, in the early analysis of kidney biopsies from people with HIVAN it was noted that often all the epithelial cells lining a renal tubule were infected with HIV-1. This phenomenon could be the result of the proliferation of renal epithelial cells within a tubule during an injury repair process [34]; if the proliferating cell is HIV+, then the majority of the cells in the repaired tubule will also contain HIV-1. An alternative explanation could be direct cell to cell spread between renal tubule cells, although we have not demonstrated that phenomenon.

Transcriptional silencing of proviral DNA, consistent with HIV latency, has also been observed in HIV-1 infected renal epithelial cells [24]. Using an in vitro HIV-1 latency model in renal tubule epithelial cells based on a dual color HIV-1 reporter virus we evaluated the effect of latency reversing agents (LRAs), both as single agents and in combination, on viral reactivation [35]. Our data showed that HIV-1 establishes latency in renal epithelial cells early post-infection and can remain latent for several weeks [35]. Treatment with LRAs induces HIV-1 reactivation only in a small fraction of latently infected cells, and the LRA combinations more effective in reactivating HIV-1 transcription in renal epithelial cells differed from those more active in T cells.

## Conclusion

ART has significantly reduced HIV-related morbidity and mortality, however HIVAN remains the third leading diagnosis in PWH with kidney disease and PWH are more likely to develop end-stage renal disease than HIV-negative individuals. HIV-1 can infect and replicate in renal epithelial cells and the expression of viral genes in those cells contributes to the development of HIVAN. To further our knowledge of HIV-1 persistence better understanding of the dynamics of HIV-1 replication, latency, cell damage and viral reactivation in these cells and other non-CD4+ T cells reservoirs is needed to inform the design of cure strategies.

## Data Availability

Not applicable.

## Abbreviations

ART: Although antiretroviral therapy
PWH: People with HIV-1
HIVAN: HIV-associated nephropathy
FSGS: Focal segmental glomerulosclerosis
HOPE: HIV Organ Policy Equity
Env: Envelope
LRAs: Latency reversing agents
